# Non-Interventional Weight Changes Are Associated with Alterations in Lipid Profiles and in the Triglyceride-to-HDL Cholesterol Ratio

**DOI:** 10.3390/nu16040486

**Published:** 2024-02-08

**Authors:** Shiri Weinstein, Elad Maor, Alon Kaplan, Tammy Hod, Avshalom Leibowitz, Ehud Grossman, Gadi Shlomai

**Affiliations:** 1 Internal Medicine D, Sheba Medical Center, Ramat Gan 5262504, Israel; shiri.weinstein@sheb.health.gov.il (S.W.); alon.kaplan@sheba.health.gov.il (A.K.); avshalom.leibowitz@sheba.health.gov.il (A.L.); 2 Faculty of Medicine, Tel-Aviv University, Tel-Aviv 6997801, Israel; elad.maor@sheba.health.gov.il (E.M.); tamar.hod@sheba.health.gov.il (T.H.); 3 Leviev Heart Center, Sheba Medical Center, Ramat Gan 5262504, Israel; 4 Renal Transplant Center, Sheba Medical Center, Ramat Gan 5262504, Israel; 5 Nephrology Department, Sheba Medical Center, Ramat Gan 5262504, Israel; 6 The Hypertension Unit, Sheba Medical Center, Ramat Gan 5262504, Israel; 7 Adelson School of Medicine, Ariel University, Ariel 407000, Israel; 8 The Institute of Endocrinology, Diabetes and Metabolism, Sheba Medical Center, Ramat Gan 5262504, Israel

**Keywords:** weight change, BMI change, lipids, triglyceride-to-HDL ratio

## Abstract

Background: Obesity is associated with dyslipidemia, and weight loss can improve obese patients’ lipid profile. Here, we assessed whether non-interventional weight changes are associated with alterations in lipid profile, particularly the triglyceride (TG)-to-high-density lipoprotein cholesterol (HDL-C) ratio (TG/HDL-C). Methods: In this retrospective analysis of subjects referred to medical screening, body mass index (BMI), low-density lipoprotein cholesterol (LDL-C), TG, and HDL-C levels were measured annually. Patients were divided according to BMI changes between visits. The primary outcomes were the changes in LDL-C, TG, HDL-C, and the TG/HDL-C ratio between visits. Results: The final analysis included 18,828 subjects. During the year of follow-up, 9.3% of the study population lost more than 5% of their weight and 9.2% gained more than 5% of their weight. The effect of weight changes on TG and on the TG/HDL-C ratio was remarkable. Patients with greater BMI increases showed greater increases in their TG/HDL-C ratio, and conversely, a decreased BMI level had lower TG/HDL-C ratios. This is true even for moderate changes of more than 2.5% in BMI. Conclusions: Non-interventional weight changes, even modest ones, are associated with significant alterations in the lipid profile. Understanding that modest, non-interventional weight changes are associated with alterations in the TG/HDL-C ratio may aid in better risk stratification and primary prevention of CV morbidity and mortality.

## 1. Introduction

Overweight and obesity are associated with an increased risk of cardiovascular (CV)-related morbidity such as hypertension (HTN), type 2 diabetes mellitus (DM), ischemic heart disease (IHD), chronic kidney disease (CKD), and dyslipidemia [1,2,3,4,5]. Dyslipidemia is an established risk factor for developing atherosclerosis and subsequent CV events [6,7,8], and interventional weight loss among overweight and obese patients improves their lipid profile [9,10,11,12]. High triglyceride (TG) levels are associated with other lipid abnormalities that predispose an individual to atherosclerosis development, including low levels of high-density lipoprotein cholesterol (HDL-C), high levels of low-density lipoprotein cholesterol (LDL-C), and atherogenic TG-rich lipoprotein remnants [13]. High TG is also associated with insulin resistance and DM, both of which are independent CV risk factors [14,15]. Insulin resistance induces the liver production and release of very low-density lipoprotein (VLDL) particles, which carry TGs in the bloodstream. Insulin resistance also decreases hepatic uptake of VLDL and LDL, resulting in increasing levels of these lipoproteins in the plasma and decreasing lipoprotein lipase activity, a major mediator of VLDL clearance [16]. While high TG levels are associated with insulin resistance and atherosclerosis [17,18,19], HDL-C promotes reverse cholesterol transport, removes excess cholesterol from arteries, and exerts anti-inflammatory and antioxidant properties [13]. In the past few years, the TG/HDL-C ratio has emerged as a surrogate marker for metabolic syndrome [20,21], increased CV risk [22,23,24,25], increased ischemic stroke risk [24,26], and CKD progression [27] and an independent predictor for all-cause mortality [28].

The effects of different dietary patterns on the lipid profile, particularly on the TG/HDL-C ratio, have been previously explored [29,30,31,32,33,34,35]. It has been reported that greater adherence to an unhealthy Western diet predicts an increased risk of abnormal blood lipids [31], that the atherogenic TG/HDL-C ratio is reduced significantly by the consumption of fresh fish [32], and that certain dietary patterns are associated with a low TG/HDL-C ratio and a lower risk for DM [30]. Furthermore, metabolic surgery in obese individuals has been shown to significantly reduce TG levels and the TG/HDL-C ratio [36,37]. However, previous studies, including recent reports, have suggested that reduced-calorie diets may result in clinically meaningful weight loss and improved glycemic control regardless of different compositions of fat, protein, and carbohydrates [38,39].

Most studies, to date, have focused on the association of various interventional weight-loss programs with specific lipid profile parameters. However, there is a paucity of data regarding the effects of non-interventional minor weight changes on the lipid profile and specifically the TG/HDL-C ratio. Most clinic patients do not participate in a structured weight loss program, yet their weight may change between clinic visits. We sought to explore the effects of any non-interventional weight changes on lipid profiles, regardless of the dietary restriction or particular nutritional habits. Specifically, we aimed to assess whether non-interventional weight changes are associated with alterations in LDL-C, HDL-C, TG, and particularly in the TG/HDL-C ratio. Our hypothesis was that even subtle weight changes may significantly affect patients’ lipid profiles.

## 2. Materials and Methods

### 2.1. Study Population

All subjects enrolled were asymptomatic men and women examined annually at the Chaim Sheba Medical Center Institute for medical screening between the years 2000 and 2020. The annual examination includes filling out a standard questionnaire regarding demographic characteristics, a complete medical history, any unusual medical events that occurred since the previous visit, and lifestyle and health-related habits. Until 2016, the electronic medical records (EMR) system included a dietary questionnaire regarding any dietary restrictions or habits, such as low-calorie diet, low-fat diet, low-carbohydrate diet, or any combination of these diets. However, with the implementation of a new EMR system, self-reported dietary restrictions are no longer available and therefore not included in our data analysis. Data regarding the dietary habits of our cohort up to 2016 are illustrated in Supplementary Figure S1. Height and weight were measured while wearing light clothing without shoes and recorded at each encounter for all participants. Participants underwent a thorough physical examination. The body mass index (BMI) was calculated as weight in kg divided by the squared height in meters. No weight loss intervention program was applied and all weight changes were subject-driven and unguided by the investigators. Blood for lab tests, including LDL-C, HDL-C, and TG levels, was drawn by a trained nurse following at least eight hours of fasting.

### 2.2. Inclusion and Exclusion Criteria

The complete database included 21,111 individuals with two consecutive annual clinic visits. Individuals were excluded if height; weight; or TG, LDL-C, or HDL-C level records were missing; or if they had extreme BMI values (less than 15 kg/m^2^ or more than 50 kg/m^2^). After patient exclusion, the final study cohort comprised 18,828 participants. Data on diet restrictions (as self-reported) were collected from 14,104 subjects.

### 2.3. Definitions and Outcome

Participants were divided according to the percent change in BMI between the first and second visits: BMI reduction of more than 5% (“large reduction”), BMI reduction between 2.5% and 5% (“moderate reduction”), BMI reduction of <2.5% or elevation of <2.5% (“unchanged”), BMI elevation between 2.5% and 5% (“moderate increase”), and BMI elevation of more than 5% (“large increase”).

The primary outcomes were changes in LDL-C, TG, HDL-C, and the TG/HDL-C ratio between visits.

### 2.4. Statistical Analysis

Trends in characteristics for categorical variables were assessed using chi-square tests. The logistic regression model was calculated to assess the relationship between the baseline characteristics of patients and increases of at least 10% in the TG/HDL-C ratio. Age, gender, initial BMI, BMI change, diagnosis of IHD, diagnosis of HTN, diagnosis of DM, and obesity (defined as BMI above 30 kg/m^2^) were tested individually and in a multivariable logistic regression model as clinically and epidemiologically relevant variables. Subset analysis was performed for gender, initial BMI, and initial TG/HDL-C ratio. All analyses were performed using R software (R Development Core Team, version 4.1.0) [40]. A 2-sided *p*-value < 0.05 was used for statistical significance.

## 3. Results

The final analysis included 18,828 patients, of whom 72% were male. Baseline demographic and clinical characteristics according to pre-specified groups of BMI change are presented in Table 1. Gender proportions of roughly two-thirds male were generally consistent across the pre-specified groups (Table 1). The mean BMI for our cohort was approximately 28 kg/m^2^ and was consistently in the overweight range across groups. Most patients in our study had a mean BMI in the overweight category (47%), followed by obesity (27%) and normal weight (26%) (Table 1). The frequency of hypertension was about 30% for the entire cohort without significant differences across groups, while mean SBP and DBP were in the range of stage 2 hypertension with a mean SBP of 126 mm Hg and a mean DBP of 79 mm Hg (Table 1). Rates of IHD and DM in our entire cohort were around 10% and were consistent across groups (Table 1).

The dietary habits of participants enrolled until the end of 2016 are illustrated in Figure S1. Of the participants, 63% indicated specific restrictions on their dietary habits. Of them, 21% self-reported adherence to a low-calorie diet, 13% to a low-fat diet, and 12% to a low-carbohydrate diet (Figure S1).

**Figure 1:**
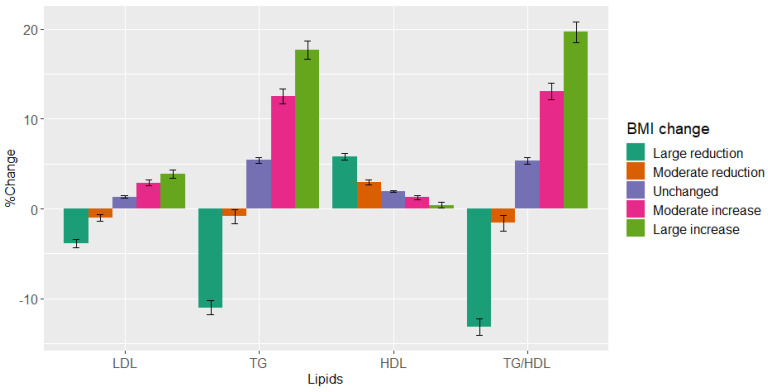
LDL-C, TG, HDL-C, and TG/HDL-C ratio change between visits across pre-specified BMI groups. Percent change in LDL-C, TG, HDL-C, and TG/HDL-C ratio between visits across pre-specified BMI groups. The analysis included 18,828 patients. *X*-axis from left to right represents LDL, TG, HDL, and TG/HDL-C ratio. *Y*-axis represents percent change in BMI. Abbreviations: LDL-C, low-density lipoprotein cholesterol; TG, triglycerides; HDL-C, high-density lipoprotein cholesterol; BMI, body mass index.

The mean baseline BMI was 26 kg/m^2^ and there was no significant change in mean BMI between visits for the entire study population (Table 2). However, 20.8% of patients reduced their BMI by at least 2.5% and 23.6% increased their BMI by more than 2.5%, and 9.3% of the patients reduced their BMI by at least 5% and 9.2% of patients increased their BMI by at least 5% (Table 2). On the second visit, patients in the pre-specified “large reduction” group had a mean percent decrease of 3.8% and 11% in LDL-C and TG, respectively, and a 5.8% increase in HDL-C (Figure 1, Table 2). Patients in the pre-specified “large increase” group had a mean percent increase of 3.9% and 18% in LDL-C and TG, respectively, and a 0.41% decrease in HDL-C (Figure 1, Table 2). The TG/HDL-C ratio decreased by 13% and increased by 20% in the pre-specified “large reduction” and “large increase” groups, respectively.

**Figure 2:**
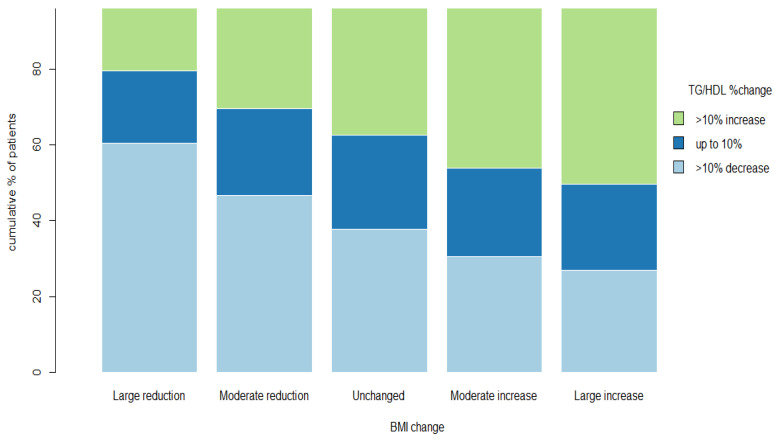
TG/HDL ratio %change according to the pre-specified BMI change. Percent change in TG/HDL-C ratio according to the pre-specified BMI change. Bars represent the pre-specified BMI change groups. Different colors represent the percent of patients in each pre-specified group with at least 10% increase in TG/HDL-C ratio from visit 1 to visit 2 (green), up to 10% change in TG/HDL-C ratio from visit 1 to visit 2 (blue) and at least 10% decrease in TG/HDL ratio from visit 1 to visit 2 (light blue). The analysis includes 18,828 patients. Abbreviations: BMI, body mass index; TG, triglycerides; HDL-C, high-density lipoprotein cholesterol.

The proportion of patients with a >10% rise in the TG/HDL-C ratio progressively increased with the relative increases in BMI, with a 20.6%, 30.2%, 37.5%, 46.2%, and 50.2% increase for the “large reduction”, “moderate reduction”, “unchanged”, “moderate increase”, and “large increase” groups, respectively (*p* < 0.01) (Figure 2). Conversely, the proportion of patients with at least a 10% decrease in the TG/HDL-C ratio progressively declined with relative decreases in BMI: 60.5%, 46.7%, 37.7%, 30.5%, and 26.9% for “large reduction”, “moderate reduction”, “unchanged”, “moderate increase”, and “large increase”, respectively (*p* < 0.01) (Figure 2).

**Figure 3:**
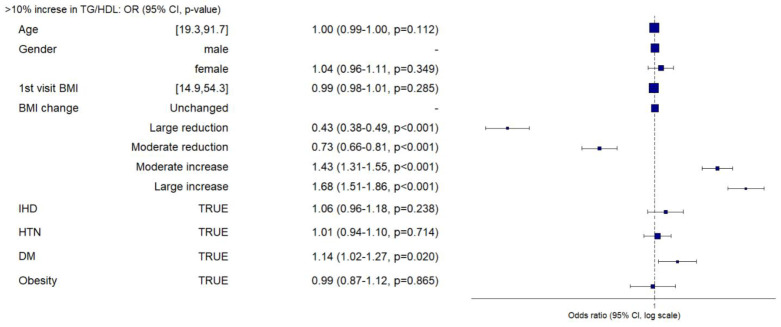
Relationship between baseline characteristics and increase of at least 10% in TG/HDL-C ratio. Logistic regression, presented as a forest plot, represents the relationship between baseline characteristics and an increase of at least 10% in the TG/HDL-C ratio on visit 2. The analysis includes 18,828 patients. Obesity is defined as BMI > 30 kg/m^2^. Abbreviations: TG, triglycerides; HDL-C, high-density lipoprotein cholesterol; BMI, body mass index; IHD, ischemic heart disease; HTN, hypertension; DM, diabetes mellitus.

In the multivariable logistic regression model, compared to the “unchanged” group, the odds ratio for TG/HDL-C ratio increases of at least 10% was 0.43, 0.73, 1.42, and 1.68 for the “large reduction”, “moderate reduction”, “moderate increase”, and “large increase” groups, respectively (*p* < 0.001) (Figure 3). Concomitant DM was significantly associated with an elevated odds ratio for a >10% rise in the TG/HDL-C ratio (*p* = 0.02). Age, gender, initial BMI, IHD diagnosis, diagnosis of HTN, and obesity, which are clinically and epidemiologic relevant variables, did not show significant correlations with the TG/HDL-C ratio increase of at least 10% in multivariable logistic regression analysis (Figure 3).

Additional analyses excluding patients who were treated with statins, and subgroup analyses by gender, showed similar results regarding the initial TG/HDL-C ratio and baseline BMI.

## 4. Discussion

In this study, we demonstrate that non-interventional weight changes are associated with significant alterations in the lipid profile. An increase in BMI, even when modest, was associated with a progressive rise in LDL-C and TG and a decrease in HDL-C. Conversely, a decrease in BMI was associated with a progressive decline in LDL-C and TG and an increase in HDL-C. The proportion of subjects with more than a 10% increase in TG/HDL-C progressively increased with a rise in BMI and conversely, patients with a decline in BMI showed a decline in their TG/HDL-C ratio.

Previous studies have shown that various interventional weight loss programs among overweight and obese individuals improve patients’ lipid profiles [9,33,36,37]. A recent retrospective study shows that among a cohort of Japanese patients, non-interventional weight losses of 5% or more are associated with improved LDL-C, TG, and HDL-C levels [41]. Our results concur with these findings. However, our cohort is much larger, and we analyzed subtler BMI changes of 2.5% or more and assessed changes in the TG/HDL-C ratio as well.

An elevated TG/HDL-C ratio has been shown to predict metabolic syndrome occurrence [21], coronary artery disease [25,42,43], peripheral artery disease [44], and cerebrovascular disease [45]. The TG/HDL-C ratio was found to positively correlate with the degree of carotid plaque vulnerability and stenosis [26]. Higher TG/HDL ratios are also associated with the prevalence of CKD and its progression [27]. Arterial stiffness is a recognized predictor of CV mortality, and death and is also associated with the TG/HDL-C ratio [46]. TG/HDL-C was a better predictor than LDL-C for atherosclerosis development [47], and relative to LDL-C, total cholesterol, HDL-C, and triglycerides, TG/HDL-C showed the strongest association with the extent of coronary disease [48]. TG/HDL-C predicted all-cause mortality and major adverse cardiac events in patients presenting for coronary angiography, even after adjusting for known cardiovascular risk factors and CAD [28]. It has been shown that even when LDL-C levels are tightly controlled, a residual CV risk remains, which might be attributable to other lipid abnormalities, such as TG/HDL-C [25,47,49]. We believe that the mentioned association of higher TG/HDL-C with different cardiovascular diseases and the predictive value of higher ratios for worse prognoses highlights the potential of TG/HDL-C to serve as a CVD risk marker.

The drawback of the TG/HDL-C ratio is the absence of a universal unequivocal cutoff value, which limits its use as a predictive biomarker for CVD [25]. Some argue that the ideal cutoff for predicting CV outcomes with the TG/HDL-C ratio is above 2, while others recommend using ratios above 2.5 and even above 3 [25,28,50]. Notably, the TG/HDL-C ratio is an important prognosticator across different weight classes and not only for overweight or obese subjects. One study found that an increased TG/HDL-C ratio correlates with a significant increase in intracerebral hemorrhage and cerebral infarction, only in patients with normal BMI [24]. Another study showed that a higher TG/HDL-C ratio was a potential risk factor for prediabetes and insulin resistance in women, even in normal-weight women [50]. Interestingly, the mean BMI for our population was almost 28 kg/m^2^, with the majority defined as either normal weight or overweight. We found that the association of non-interventional weight changes and alterations in lipid profile was independent of baseline BMI as well as gender.

In our cohort population, concomitant DM was significantly associated with an increased odds ratio of a ≥10% rise in the TG/HDL-C ratio. This finding is likely related to insulin resistance-mediated increased TG production by the liver and reduced clearance of TG-rich lipoproteins from the bloodstream [16] and concurs with the current literature, as the association between the TG/HDL-C ratio and insulin resistance has been widely explored. It has been shown that a high plasma TG/HDL-C ratio provides a simple means of identifying insulin-resistant obese patients who are likely to have increased risks for CVD [16,20,51,52]. In addition, the TG/HDL-C ratio significantly correlates with fasting insulin levels even among adults without DM [53], and the TG/HDL-C ratio is a valuable predictor of DM incidence [54]. Our findings highlight the importance of even minor weight changes, specifically among patients with DM, and suggest that individuals with DM who gain weight may be at a higher risk of worsening dyslipidemia and CVD development.

The TG/HDL-C ratio is potentially a modifiable risk factor as some studies suggest. It has been shown that specific dietary patterns obtained by a validated food frequency questionnaire are associated with changes in the TG/HDL-C ratio and ultimately with a reduced risk for DM [30]. In addition, in a randomized open-labeled trial, the consumption of fresh fish significantly reduced the TG/HDL-C ratio among patients with hyperlipidemia [32], and finally, a prospective cohort found that a greater adherence to the Western diet pattern increased the TG/HDL-C ratio and predicted a higher risk for abnormal lipids overall [31]. However, while the effect of different dietary patterns on lipid composition has been established, the influence of various diet compositions on weight loss is somewhat controversial. A large randomized trial showed that reduced-calorie diets result in clinically meaningful weight loss regardless of which macronutrients are consumed [33]. Other investigators have shown that the difference found in the effect of calorie-unrestricted low-carbohydrate/high-fat diets versus high-carbohydrate/low-fat diets on glycemic control and weight loss was not sustained three months after intervention [55]. Furthermore, in a recent study, a comparison of weight reduction using a very-low-carbohydrate diet versus a low-carbohydrate diet in obese patients yielded no significantly different outcomes, such as body weight and fat, lipid abnormalities, and liver function [34].

The aim of our study was to assess the association of non-interventional weight changes on the lipid profile and particularly, the TG/HDL-C ratio, regardless of any dietary habits or restrictions. Most patients do not participate in structured weight loss programs, yet we frequently observed modest weight changes, increases or decreases, between clinic visits. Therefore, we believed it to be prudent to evaluate the effects of these seemingly negligible weight changes on specific lipid parameters in this specific and very large proportion of clinic patients. Our findings, together with data from interventional weight programs, further highlight the importance of weight changes on the lipid profile and possibly on CV health.

One of our study’s limitations is the lack of individual dietary data for our total cohort, which is attributed to the change in our EMR system from 2016. To partially overcome this limitation and to provide a general idea of our study population’s dietary habits, we conducted an analysis of approximately 14,000 patients who were assessed in the same medical screening facility prior to 2016 and for whom dietary data exist. Our study has several other limitations. First, this is a retrospective study. Therefore, causality between BMI alterations and serum lipids could not be established. Second, data regarding the method for any weight changes obtained, including physical activity, are not available. In addition, our cohort had a clear male predominance (72%), which might reduce the generalization of our findings. However, in a subgroup analysis, we did not observe any gender-related significant differences in the association between weight changes and lipid profile.

## 5. Conclusions

In conclusion, we provide evidence that non-interventional weight changes, even when modest and subtle, are associated with changes in the lipid profile and in the TG/HDL-C ratio. Our findings concur with previous reports regarding the association between weight loss and alterations in lipid profile, specifically in the TG/HDL-C ratio.

Early detection of elevated TG/HDL-C ratios may serve as a strategy to prevent the development of atherosclerotic complications, especially in high-risk populations. Understanding that alterations in the TG/HDL-C ratio may be associated with modest, non-interventional weight changes may aid in better risk stratification and primary prevention of CV morbidity and mortality. Larger-scale prospective studies, with individual dietary data and long-term follow-up, are pivotal in evaluating and understanding the significance of modest non-interventional weight changes on CV morbidity and mortality.

## Figures and Tables

**Table 1:** Baseline characteristics.

	Large Reduction	Moderate Reduction	Unchanged	Moderate Increase	Large Increase	Total	*p* Value
	(*N* = 1754)	(*N* = 2163)	(*N* = 10,524)	(*N* = 2664)	(*N* = 1723)	(*N* = 18,828)	
Gender							<0.001
Males	1169 (67%)	1592 (74%)	7852 (75%)	1944 (73%)	1075 (62%)	13,632 (72%)	
Females	585 (33%)	571 (26%)	2672 (25%)	720 (27%)	648 (38%)	5196 (28%)	
Age (years)							<0.001
Mean (SD)	48.9 (±10.3)	50.4 (±10.7)	50.0 (±10.3)	49.2 (±10.4)	48.1 (±9.9)	49.7 (±10.4)	
1st visit BMI (kg/m^2^)							<0.001
Mean (SD)	27.9 (±4.5)	26.4 (±3.6)	25.8 (±3.7)	25.6 (±3.8)	25.3 (±4.0)	26.0 (±3.8)	
BMI categories							<0.001
<18.5	5 (0%)	9 (0%)	109 (1%)	19 (1%)	30 (2%)	172 (1%)	
18.5–25	452 (26%)	788 (36%)	4547 (43%)	1308 (49%)	843 (49%)	7938 (42%)	
25–30	832 (47%)	1045 (48%)	4633 (44%)	1020 (38%)	653 (38%)	8183 (43%)	
>30	465 (27%)	321 (15%)	1235 (12%)	317 (12%)	197 (11%)	2535 (13%)	
IHD	200 (11%)	266 (12%)	1251 (12%)	285 (11%)	166 (10%)	2168 (12%)	0.0331
SBP (mm Hg)							<0.001
Mean (SD)	125.9 (±18.4)	124.7 (±17.7)	124.2 (±17.3)	122.9 (±16.7)	121.4 (±16.6)	124.0 (±17.3)	
DBP (mm Hg)							<0.001
Mean (SD)	79.1 (±11.1)	78.1 (±10.4)	77.7 (±10.4)	77.3 (±10.2)	76.3 (±10.5)	77.7 (±10.5)	
HTN	501 (29%)	667 (31%)	3048 (29%)	725 (27%)	489 (28%)	5430 (29%)	0.0934
DM	182 (10%)	220 (10%)	981 (9%)	256 (10%)	150 (9%)	1789 (10%)	0.356

Abbreviations: BMI, body mass index; IHD, ischemic heart disease; SBP, systolic blood pressure; DBP, diastolic blood pressure; HTN, hypertension; DM, diabetes mellitus.

**Table 2:** Body mass index and lipid levels.

	Large Reduction	Moderate Reduction	Unchanged	Moderate Increase	Large Increase	Total	*p* Value
	(*N* = 1754)	(*N* = 2163)	(*N* = 10,524)	(*N* = 2664)	(*N* = 1723)	(*N* = 18,828)	
1st visit BMI (kg/m^2^)							<0.001
Mean (SD)	27.9 (±4.5)	26.4 (±3.6)	25.8 (±3.7)	25.5 (±3.8)	25.3 (±4.0)	26.0 (±3.8)	
2nd visit BMI (kg/m^2^)							<0.001
Mean (SD)	25.4 (±3.8)	25.4 (±3.5)	25.8 (±3.7)	26.5 (±3.9)	27.3 (±4.4)	25.9 (±3.8)	
%BMI change							<0.001
Mean (SD)	−8.6 (±4.2)	−3.6 (±0.7)	0.1 (±1.3)	3.6 (±0.7)	8.0 (±4.7)	0.1 (±4.5)	
1st visit LDL (mg/dL)							<0.001
Mean (SD)	123.5 (±30.0)	122.4 (±28.2)	121.5 (±28.2)	120.0 (±27.1)	120.4 (±29.4)	121.5 (±28.4)	
2nd visit LDL (mg/dL)							<0.001
Mean (SD)	116.5 (±28.8)	119.4 (±28.1)	121.3 (±28.0)	121.7 (±27.2)	122.8 (±29.1)	120.8 (±28.1)	
%LDL change							<0.001
Mean (SD)	−3.8 (±20.1)	−1.0 (±17.3)	1.3 (±17.4)	2.9 (±17.0)	3.9 (±18.6)	1.0 (±17.8)	
1st visit HDL (mg/dL)							<0.001
Mean (SD)	47.1 (±12.3)	47.3 (±12.0)	48.2 (±12.5)	48.3 (±12.0)	49.9 (±13.4)	48.2 (±12.4)	
2nd visit HDL (mg/dL)							0.0046
Mean (SD)	49.3 (±12.6)	48.3 (±11.9)	48.7 (±12.3)	48.5 (±11.8)	49.6 (±13.0)	48.8 (±12.3)	
%HDL change							<0.001
Mean (SD)	5.8 (±15.4)	3.0 (±12.8)	1.9 (±12.3)	1.3 (±12.3)	0.4 (±13.2)	2.2 (±12.8)	
1st visit TG (mg/dL)							<0.001
Mean (SD)	135.1 (±71.1)	130.0 (±67.5)	124.3 (±62.9)	118.9 (±59.4)	115.8 (±59.4)	124.4 (±63.7)	
2nd visit TG (mg/dL)							<0.001
Mean (SD)	111.0 (±57.0)	120.3 (±60.1)	124.9 (±63.3)	127.6 (±65.4)	129.9 (±68.5)	123.9 (±63.4)	
%TG change							<0.001
Mean (SD)	−11.0 (±32.6)	−0.8 (±36.6)	5.4 (±33.7)	12.5 (±44.2)	17.7 (±42.7)	5.3 (±37.2)	
1st visit TG/HDL-C ratio							<0.001
Mean (SD)	3.2 (±2.2)	3.1 (±2.1)	2.9 (±1.9)	2.7 (±1.8)	2.6 (±1.8)	2.9 (±2.0)	
2nd visit TG/HDL-C ratio							<0.001
Mean (SD)	2.5 (±1.7)	2.8 (±1.8)	2.9 (±1.9)	2.9 (±1.9)	2.9 (±2.0)	2.8 (±1.9)	
TG_HDL_ratio_change							<0.001
Mean (SD)	−13.2 (±37.8)	−1.6 (±40.5)	5.3 (±37.9)	13.0 (±47.8)	19.7 (±47.9)	5.2 (±41.5)	

Abbreviations: BMI, body mass index; TG, triglycerides; HDL-C, high-density lipoprotein cholesterol; LDL, low-density lipoprotein.

## Data Availability

The data presented in this study are available on request from the corresponding author. The data are not publicly available due to privacy reasons.

## Supplementary Materials

The following supporting information can be downloaded at https://www.mdpi.com/article/10.3390/nu16040486/s1, Figure S1: Pie chart representing the proportion of specific dietary restrictions and habits of 14,104 subjects, who were assessed in the same medical screening facility prior to 2016 and for whom dietary data exist.

## References

[1] Piché M.E., Tchernof A., Després J.P. (2020). Obesity Phenotypes, Diabetes, and Cardiovascular Diseases. Circ. Res..

[2] Leggio M., Lombardi M., Caldarone E., Severi P., D’emidio S., Armeni M., Bravi V., Bendini M.G., Mazza A. (2017). The relationship between obesity and hypertension: An updated comprehensive overview on vicious twins. Hypertens. Res..

[3] Wormser D., Kaptoge S., Di Angelantonio E., Wood A.M., Pennells L., Thompson A., Sarwar N., Kizer J.R., Lawlor D.A., Nordestgaard B.G. (2011). Separate and combined associations of body-mass index and abdominal adiposity with cardiovascular disease: Collaborative analysis of 58 prospective studies. Lancet.

[4] Hsu C.Y., McCulloch C.E., Iribarren C., Darbinian J., Go A.S. (2006). Body mass index and risk for end-stage renal disease. Ann. Intern. Med..

[5] Al Rifai M., Silverman M.G., Nasir K., Budoff M.J., Blankstein R., Szklo M., Katz R., Blumenthal R.S., Blaha M.J. (2015). The association of nonalcoholic fatty liver disease, obesity, and metabolic syndrome, with systemic inflammation and subclinical atherosclerosis: The Multi-Ethnic Study of Atherosclerosis (MESA). Atherosclerosis.

[6] Koenen M., Hill M.A., Cohen P., Sowers J.R. (2021). Obesity, Adipose Tissue and Vascular Dysfunction. Circ. Res..

[7] Navar-Boggan A.M., Peterson E.D., D’Agostino R.B., Neely B., Sniderman A.D., Pencina M.J. (2015). Hyperlipidemia in early adulthood increases long-term risk of coronary heart disease. Circulation.

[8] Kaneko H., Itoh H., Kiriyama H., Kamon T., Fujiu K., Morita K., Michihata N., Jo T., Takeda N., Morita H. (2021). Lipid Profile and Subsequent Cardiovascular Disease among Young Adults Aged <50 Years. Am. J. Cardiol..

[9] Jiménez J.-M., Carbajo M.-A., López M., Cao M.-J., Rúiz-Tovar J., García S., Castro M.-J. (2020). Changes in Lipid Profile, Body Weight Variables and Cardiovascular Risk in Obese Patients Undergoing One-Anastomosis Gastric Bypass. Int. J. Environ. Res. Public Health.

[10] Noakes M., Clifton P.M. (2000). Weight loss and plasma lipids. Curr. Opin. Lipidol..

[11] Grundy S.M. (2016). Metabolic syndrome update. Trends Cardiovasc. Med..

[12] Hasan B., Nayfeh T., Alzuabi M., Wang Z., Kuchkuntla A.R., Prokop L.J., Newman C.B., Murad M.H., Rajjo T.I. (2020). Weight Loss and Serum Lipids in Overweight and Obese Adults: A Systematic Review and Meta-Analysis. J. Clin. Endocrinol. Metab..

[13] Welty F.K. (2013). How Do Elevated Triglycerides and Low HDL-Cholesterol Affect Inflammation and Atherothrombosis?. Curr. Cardiol. Rep..

[14] Kosmas C.E., Bousvarou M.D., Kostara C.E., Papakonstantinou E.J., Salamou E., Guzman E. (2023). Insulin resistance and cardiovascular disease. J. Int. Med. Res..

[15] Wong N.D., Sattar N. (2023). Cardiovascular risk in diabetes mellitus: Epidemiology, assessment and prevention. Nat. Rev. Cardiol..

[16] Ormazabal V., Nair S., Elfeky O., Aguayo C., Salomon C., Zuñiga F.A. (2018). Association between insulin resistance and the development of cardiovascular disease. Cardiovasc. Diabetol..

[17] Grundy S.M. (1999). Hypertriglyceridemia, insulin resistance, and the metabolic syndrome. Am. J. Cardiol..

[18] Abbasi F., Kohli P., Reaven G.M., Knowles J.W. (2016). Hypertriglyceridemia: A simple approach to identify insulin resistance and enhanced cardio-metabolic risk in patients with prediabetes. Diabetes Res. Clin. Pract..

[19] Kraegen E.W., Cooney G.J., Ye J., Thompson A.L. (2001). Triglycerides, fatty acids and insulin resistance–hyperinsulinemia. Exp. Clin. Endocrinol. Diabetes.

[20] McLaughlin T., Abbasi F., Cheal K., Chu J., Lamendola C., Reaven G. (2003). Use of metabolic markers to identify overweight individuals who are insulin resistant. Ann. Intern. Med..

[21] Abbasian M., Delvarianzadeh M., Ebrahimi H., Khosravi F. (2017). Lipid ratio as a suitable tool to identify individuals with MetS risk: A case- control study. Diabetes Metab. Syndr..

[22] Shao Q.-Y., Ma X.-T., Yang Z.-Q., Li Q.-X., Wang Y.-F., Liang J., Shen H., Liu X.-L., Zhou Y.-J., Shi D.-M. (2022). Prognostic significance of multiple triglycerides-derived metabolic indices in patients with acute coronary syndrome. J. Geriatr. Cardiol..

[23] Wang B., Hua J., Ma L. (2022). Triglyceride to High-Density Lipoprotein Ratio can predict coronary artery calcification. Pakistan J. Med. Sci..

[24] Sato F., Nakamura Y., Kayaba K., Ishikawa S. (2022). TG/HDL-C ratio as a predictor of stroke in the population with healthy BMI: The Jichi Medical School Cohort Study. Nutr. Metab. Cardiovasc. Dis..

[25] Kosmas C.E., Rodriguez Polanco S., Bousvarou M.D., Papakonstantinou E.J., Peña Genao E., Guzman E., Kostara C.E. (2023). The Triglyceride/High-Density Lipoprotein Cholesterol (TG/HDL-C) Ratio as a Risk Marker for Metabolic Syndrome and Cardiovascular Disease. Diagnostics.

[26] Zhao Z., Wang H., Hou Q., Zhou Y., Zhang Y. (2023). Non-traditional lipid parameters as potential predictors of carotid plaque vulnerability and stenosis in patients with acute ischemic stroke. Neurol. Sci..

[27] Nguyen H.H., Tran H.H., Nguyen L.T., Nguyen T., Nguyen N.A., Vi M.T., Nguyen K.T. (2022). TG/HDL-C Ratio Is a Risk Factor Associated with CKD: Use in Assessing the Risk of Progression of CKD. Pathophysiology.

[28] Sultani R., Tong D.C., Peverelle M., Lee Y.S., Baradi A., Wilson A.M. (2020). Elevated Triglycerides to High-Density Lipoprotein Cholesterol (TG/HDL-C) Ratio Predicts Long-Term Mortality in High-Risk Patients. Heart. Lung Circ..

[29] Fechner E., Smeets E.T.H.C., Schrauwen P., Mensink R.P. (2020). The Effects of Different Degrees of Carbohydrate Restriction and Carbohydrate Replacement on Cardiometabolic Risk Markers in Humans-A Systematic Review and Meta-Analysis. Nutrients.

[30] Song S., Lee J.E. (2018). Dietary Patterns Related to Triglyceride and High-Density Lipoprotein Cholesterol and the Incidence of Type 2 Diabetes in Korean Men and Women. Nutrients.

[31] Ushula T.W., Mamun A., Darssan D., Wang W.Y.S., Williams G.M., Whiting S.J., Najman J.M. (2022). Dietary patterns and the risk of abnormal blood lipids among young adults: A prospective cohort study. Nutr. Metab. Cardiovasc. Dis..

[32] Zibaeenezhad M.J., Ghavipisheh M., Attar A., Aslani A. (2017). Comparison of the effect of omega-3 supplements and fresh fish on lipid profile: A randomized, open-labeled trial. Nutr. Diabetes.

[33] Sacks F.M., Bray G.A., Carey V.J., Smith S.R., Ryan D.H., Anton S.D., McManus K., Champagne C.M., Bishop L.M., Laranjo N. (2009). Comparison of weight-loss diets with different compositions of fat, protein, and carbohydrates. N. Engl. J. Med..

[34] Kikuchi T., Kushiyama A., Yanai M., Kashiwado C., Seto T., Kasuga M. (2023). Comparison of Weight Reduction, Change in Parameters and Safety of a Very Low Carbohydrate Diet in Comparison to a Low Carbohydrate Diet in Obese Japanese Subjects with Metabolic Disorders. Nutrients.

[35] Ma C., Avenell A., Bolland M., Hudson J., Stewart F., Robertson C., Sharma P., Fraser C., MacLennan G. (2017). Effects of weight loss interventions for adults who are obese on mortality, cardiovascular disease, and cancer: Systematic review and meta-analysis. BMJ.

[36] Santos J., Salgado P., Santos C., Mendes P., Saavedra J., Baldaque P., Monteiro L., Costa E. (2014). Effect of bariatric surgery on weight loss, inflammation, iron metabolism, and lipid profile. Scand. J. Surg..

[37] Wang W., Fann C.S.J., Yang S.-H., Chen H.-H., Chen C.-Y. (2019). Weight loss and metabolic improvements in obese patients undergoing gastric banding and gastric banded plication: A comparison. Nutrition.

[38] Unick J.L., Beavers D., Jakicic J.M., Kitabchi A.E., Knowler W.C., Wadden T.A., Wing R.R., Look AHEAD Research Group (2011). Effectiveness of lifestyle interventions for individuals with severe obesity and type 2 diabetes: Results from the Look AHEAD trial. Diabetes Care.

[39] Ayisi Addo S., Nti C., Vuvor F., Adjimani J., Steiner-Asiedu M. (2019). Impact of Successful Weight Loss Maintenance on Serum Lipids and Glucose Concentrations of Previous Participants of a Weight Loss Programme in Accra, Ghana. J. Nutr. Metab..

[40] R Core Team (2022). R: A Language and Environment for Statistical Computing.

[41] Kiriyama H., Kaneko H., Itoh H., Kamon T., Mizuno Y., Fujiu K., Morita H., Yamamichi N., Komuro I. (2021). Association between changes in body weight and lipid profile in the general population: A community-based cohort study. Eur. Heart J. Qual. Care Clin. Outcomes.

[42] Park B., Jung D.H., Lee H.S., Lee Y.J. (2021). Triglyceride to HDL-Cholesterol Ratio and the Incident Risk of Ischemic Heart Disease Among Koreans Without Diabetes: A Longitudinal Study Using National Health Insurance Data. Front. Cardiovasc. Med..

[43] Martínez-Marroquín Y., Meaney A., Samaniego-Méndez V., Nájera N., Ceballos G., Fernández-Barros C., Meaney E. (2023). The TG/HDL-c Lipid Ratio as a Cardiovascular Risk Marker in a Mexican Urban Middle-Class Population: Do We Need a Risk Score Tailored for Mexicans?. J. Clin. Med..

[44] Mesut E., Cihan A., Orhan G. (2020). Is it possible to predict the complexity of peripheral artery disease with atherogenic index?. Vascular.

[45] Gu X., Li Y., Chen S., Yang X., Liu F., Li Y., Li J., Cao J., Liu X., Chen J. (2019). Association of Lipids with Ischemic and Hemorrhagic Stroke: A Prospective Cohort Study among 267,500 Chinese. Stroke.

[46] Baba M., Maris M., Jianu D., Luca C.T., Stoian D., Mozos I. (2023). The Impact of the Blood Lipids Levels on Arterial Stiffness. J. Cardiovasc. Dev. Dis..

[47] Edwards M.K., Blaha M.J., Loprinzi P.D. (2017). Atherogenic Index of Plasma and Triglyceride/High-Density Lipoprotein Cholesterol Ratio Predict Mortality Risk Better than Individual Cholesterol Risk Factors, among an Older Adult Population. Mayo Clin. Proc..

[48] da Luz P.L., Favarato D., Faria-Neto J.R., Lemos P., Chagas A.C.P. (2008). High ratio of triglycerides to HDL-cholesterol predicts extensive coronary disease. Clinics.

[49] Dhindsa D.S., Sandesara P.B., Shapiro M.D., Wong N.D. (2020). The Evolving Understanding and Approach to Residual Cardiovascular Risk Management. Front. Cardiovasc. Med..

[50] Borrayo G., Basurto L., González-Escudero E., Diaz A., Vázquez A., Sánchez L., Hernández-González G.O., Barrera S., Degollado J.A., Córdova N. (2018). Tg/Hdl-C Ratio as Cardio-Metabolic Biomarker even in Normal Weight Women. Acta Endocrinol..

[51] McLaughlin T., Reaven G., Abbasi F., Lamendola C., Saad M., Waters D., Simon J., Krauss R.M. (2005). Is there a simple way to identify insulin-resistant individuals at increased risk of cardiovascular disease?. Am. J. Cardiol..

[52] Salazar M.R., Carbajal H.A., Espeche W.G., Leiva Sisnieguez C.E., Balbín E., Dulbecco C.A., Aizpurúa M., Marillet A.G., Reaven G.M. (2012). Relation among the plasma triglyceride/high-density lipoprotein cholesterol concentration ratio, insulin resistance, and associated cardio-metabolic risk factors in men and women. Am. J. Cardiol..

[53] Li C., Ford E.S., Meng Y.-X., Mokdad A.H., Reaven G.M. (2008). Does the association of the triglyceride to high-density lipoprotein cholesterol ratio with fasting serum insulin differ by race/ethnicity?. Cardiovasc. Diabetol..

[54] Vega G.L., Barlow C.E., Grundy S.M., Leonard D., DeFina L.F. (2014). Triglyceride-to-high-density-lipoprotein-cholesterol ratio is an index of heart disease mortality and of incidence of type 2 diabetes mellitus in men. J. Investig. Med..

[55] Hansen C.D., Gram-Kampmann E.-M., Hansen J.K., Hugger M.B., Madsen B.S., Jensen J.M., Olesen S., Torp N., Rasmussen D.N., Kjærgaard M. (2023). Effect of Calorie-Unrestricted Low-Carbohydrate, High-Fat Diet Versus High-Carbohydrate, Low-Fat Diet on Type 2 Diabetes and Nonalcoholic Fatty Liver Disease: A Randomized Controlled Trial. Ann. Intern. Med..

