# Supplementary figures and images for: Non-Interventional Weight Changes Are Associated with Alterations in Lipid Profiles and in the Triglyceride-to-HDL Cholesterol Ratio
Source: Nutrients. 2024 Feb 8;16(4):486. doi: 10.3390/nu16040486 (PMC10892159; doi:10.3390/nu16040486)

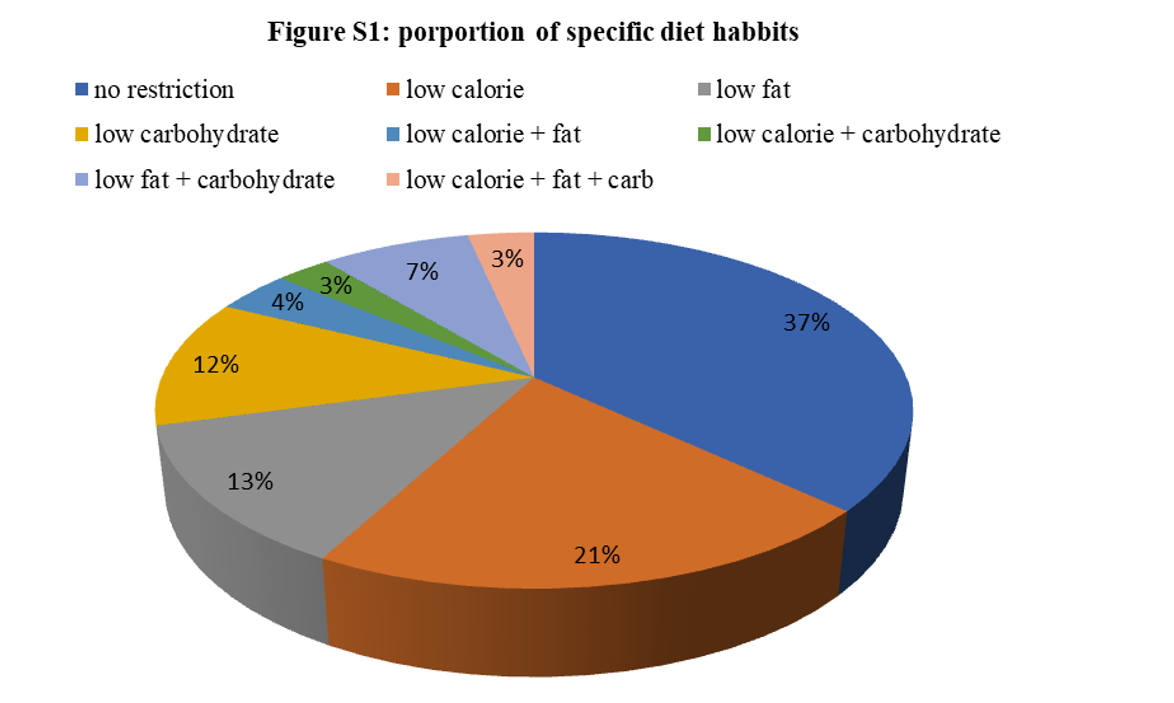

Supplement: Supplementary file 1 [file nutrients-16-00486-s001.zip › nutrients-2824420-supplementary.png]
